# Treatment of Chylothorax with Pleurodesis (A Lesser Known Complication of Behçet's Disease): A Case Report

**Published:** 2018-10

**Authors:** Ertan Demirdaş, Kıvanç Atilgan, Zafer Cengiz ER, Süleyman Emre Akin

**Affiliations:** 1 *Department of Cardiovascular Surgery, Faculty of Medicine, Bozok University, Yozgat, Turkey.*; 2 *Yozgat State Hospital, Yozgat, Turkey.*

**Keywords:** *Chylothorax*, *Behcet syndrome*, *Vasculitis*, *Thrombosis*

## Abstract

Behçet's Disease (BD) is a multisystemic vasculitis which usually affects optical, genital, and oral mucosae and often reoccurs intermittently. Chylothorax is a very rare complication of BD which usually causes thrombosis in the major venous system. A 27-year-old man with a 10-year history of BD referred to our cardiovascular surgery department with symptoms of serious aches in the left arm, edema, and apparent veins on the left anterior chest wall. A total thrombosis of the left internal and external jugular veins and the left subclavian vein was observed. One month after a successful treatment and discharge, the patient returned to our clinic with symptoms of dyspnea and coughs. A chest radiograph showed a consolidated region. A milky liquid was aspirated through thoracocentesis from the left thorax, and its biochemical analysis helped us arrive at a diagnosis of chylothorax. The patient was hospitalized and administered corticosteroids and immunosuppressive therapy with a high-carbohydrate and low-fat dietary regimen for BD. Thereafter, a left thoracic drainage system was established. On the seventh day of hospitalization, due to a progressing cheilosis flow, a pleurodesis process was applied with talcum powder. However, the chylous drainage was continued and 60 mL of venous autologous blood was injected into the left thorax through a drainage tube. The treatment was successful, and the patient was discharged from the hospital uneventfully. At 1 month’s follow-up, the chest radiograph was normal.

## Introduction

Behçet's Disease (BD), which has an autoimmune pathophysiology, is a multisystemic vasculitis that usually reoccurs intermittently and affects optical, genital, and oral mucosae.^1^^-^^4^ Although occasionally detected in various parts of the world, BD is seen more frequently in the Middle East, the Mediterranean region, and the Far East Asian countries.^3^^-^^5^ Chylothorax is a very rare complication of BD which usually causes thrombosis in the major venous system.^2^

## Case Report

A 27-year-old man with a 10-year history of BD, which had been left untreated for the preceding 2 years, referred to our cardiovascular surgery department with symptoms of serious aches in the left arm, edema, and apparent veins on the left anterior chest wall. The color Doppler ultrasonography of the left upper extremity venous system showed a total thrombosis of the left internal and external jugular veins and the left subclavian vein.

**Figure 1:**
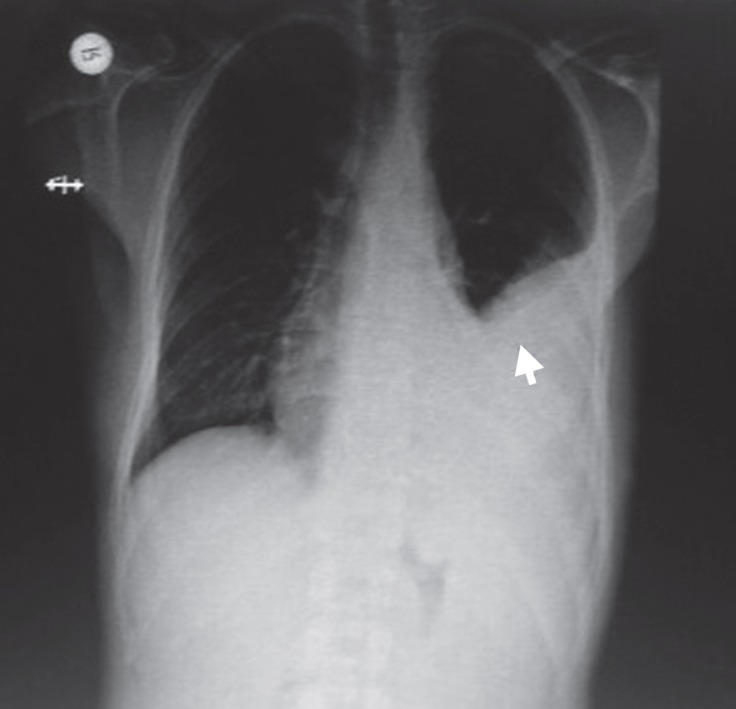
Posteroanterior chest radiography, on admission, shows a consolidated region in the left thoracic cavity (arrow).

A single dose of low molecular weight heparin (LMWH) (7500 IU/0.3 mL of bemiparin sodium) treatment for a day, antibiotics, and anti-inflammatory drugs were ordered. One month after the hospital discharge, the patient returned to our clinic with symptoms of dyspnea and coughs. A physical examination revealed decreased occultation sounds in the middle and bottom fields of the left thorax. A posteroanterior direct chest radiograph illustrated a consolidated region, in line with the auscultation findings (Figure 1). A milky liquid was aspirated via thoracocentesis from the left thorax and was subsequently biochemically analyzed; the results demonstrated total protein of 4.3 g/dL, albumin of 2.2 g/dL, total cholesterol of 47 mg/dL, and triglycerides of 287 mg/dL.

**Figure 2:**
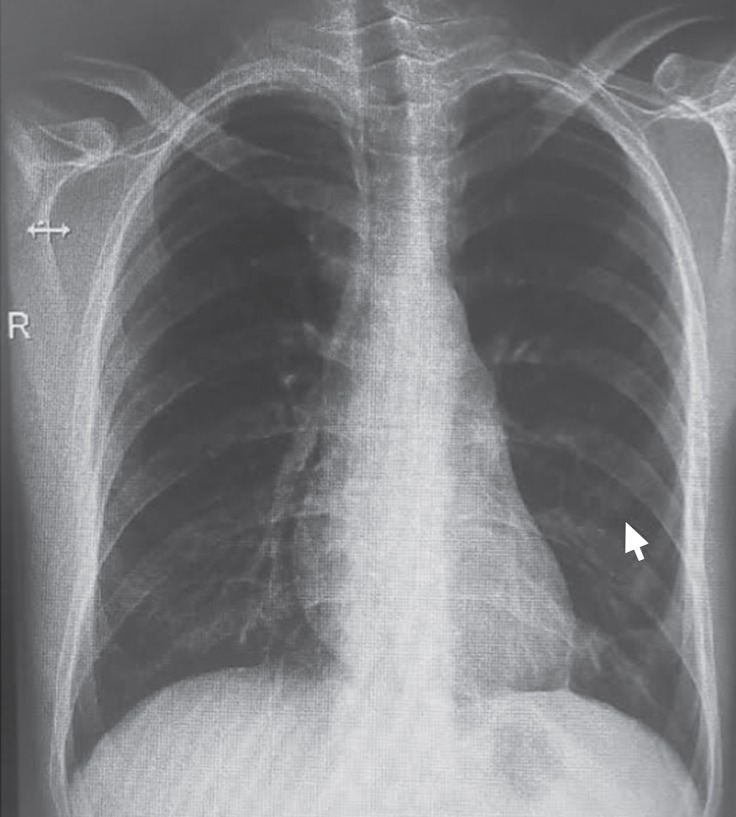
Control posteroanterior radiography, 1 month after the hospital discharge, shows the total resolution of the consolidated region (arrow).

In light of the findings, a diagnosis of chylothorax was established. The patient was hospitalized and received single doses of LMWH, 0.5 mg of colchicine, 40 mg of an oral corticosteroid (prednisolone), 2 doses of oral immunosuppressive therapy (50 mg of azathioprine), and a high-carbohydrate and low-fat dietary regimen. A left thoracic drainage system was established for the patient. On the seventh day of hospitalization, due to a progressing cheilosis flow, a pleurodesis process was applied with talcum powder. However, the chylothorax progression continued for the following 3 days. Venous autologous blood (60 mL) was injected into the left thorax via a drainage tube, which was clamped in order to avoid the leakage of the blood back through it. For the ensuing 3 hours, the patient was moved into different lying positions. Two days later, the cheilosis flow decreased to an acceptable level, and the dietary limitations were canceled. At the end of the 15th day following the autologous blood pleurodesis process, the cheilosis flow totally disappeared. The patient was discharged with a treatment of an oral anticoagulant (5 mg of warfarin) and a single dose of an oral immunosuppressive therapy (50 mg of azathioprine). One month after the hospital discharge, no pathology was observed on a posteroanterior direct chest radiogram (Figure 2).

## Discussion

An inflammatory, multisystemic, and chronic vasculitis, BD can affect the optical, genital, and oral mucosae in the human body independently of size.^6^ The venous system is more vulnerable than the arterial system in BD.^7^

Chylothorax can be defined as the collection of a lymphatic liquid in the pleural space for any reason. The thoracic duct provides the lymphatic drainage of the cheilosis liquid, most of which comes from the intestines and drains into the venous system at the junction of the left subclavian and left jugular veins. Any obstruction at this junctional area may result in chylothorax^8^ In BD, the thrombosis of the superior vena cava, the innominate vein, and the left subclavian vein may cause a disorder in the lymphatic drainage, which may lead to chylothorax.^9^

In the treatment of chylothorax related to BD, less invasive techniques should be preferred. These techniques basically are pleural effusion drainage, anticoagulant therapy for thrombosis, oral corticosteroids, immunosuppressive treatments, and high-carbohydrate and low-fat dietary regimens. In resistant cases, pleurodesis or the ligation of the thoracic duct via videothoracoscopy and thoracotomy should be preferred.^10^^-^^12^

In this case, we managed to treat a chylothorax related to BD with autologous blood pleurodesis.

## Conclusion

Chylothorax related to BD, albeit a rare complication, is possible to cure with both medical and invasive techniques.

## Notes:

***This paper should be cited as: ***Demirdaş E, Atilgan K, Cengiz ER Z, Emre Akin S. Treatment of Chylothorax with Pleurodesis (A Lesser Known Complication of Behçet's Disease): A Case Report. J Teh Univ Heart Ctr 2018;13(4):180-182.
